# Telitacicept for refractory AChR-positive generalized myasthenia gravis: a retrospective real-world study

**DOI:** 10.3389/fimmu.2026.1752245

**Published:** 2026-02-09

**Authors:** Xi Rong, Xupeng Sun, Li Wang, Meijie Qu, Liwei Jiang, Min Liu

**Affiliations:** 1 Department of Neurology, The Affiliated Hospital of Qingdao University, Qingdao, China; 2 Department of Otolaryngology-Head and Neck Surgery, The Affiliated Hospital of Qingdao University, Qingdao, China

**Keywords:** acetylcholine receptor, B-cell activating factor, myasthenia gravis, real-world evidence, refractory generalized myasthenia gravis, telitacicept

## Abstract

**Background and aims:**

Treating refractory acetylcholine receptor-positive generalized myasthenia gravis (AChR+ gMG) remains challenging, especially for patients requiring long-term immunosuppressive therapy. Current treatments often lack specificity and pose significant long-term risks, underscoring the need for alternatives. Telitacicept, a novel dual inhibitor of B lymphocyte stimulator (BLyS) and proliferation-inducing ligand (APRIL), offers a promising targeted therapeutic approach. This study aimed to evaluate the efficacy and safety of telitacicept in the treatment of refractory AChR+ generalized myasthenia gravis.

**Methods:**

This retrospective study included 42 patients with refractory AChR+ gMG who received telitacicept. The primary outcomes assessed were changes from baseline in Quantitative Myasthenia Gravis (QMG) scores, analyzed using mixed-effects models. Secondary outcomes comprised cumulative response rates, reductions in concomitant immunosuppressive medications, and safety events.

**Results:**

A total of 42 refractory MG patients with MGFA class II–IV were enrolled. Significant improvements were observed in the QMG total score (least-squares [LS] mean change at month 5: -2.24, 95% CI -3.34 to -1.15, p<0.001), with sustained benefits across ocular, limb, and bulbar areas. Cumulative response rates reached 69.9% for MGFA-PIS and 73.8% for QMG improvement (≥3-point reduction) by 6 months. Notable decreases in prednisone (LS mean -10.17 mg/day, p<0.001) and immunosuppressant use were also seen. The therapy demonstrated a promising safety profile.

**Conclusions:**

Telitacicept demonstrated significant efficacy in refractory AChR+ gMG and may reduce dependence on traditional immunosuppressants. These real-world findings support its use as a valuable treatment choice for this challenging patient group.

## 1 Introduction

Myasthenia gravis (MG) is a heterogeneous autoimmune disorder caused by pathogenic antibodies, characterized by muscle weakness and fatigue. It mainly affects the neuromuscular junction, where autoantibodies target proteins essential for muscle contraction, disrupting communication between nerves and muscles. The disease is categorized into subgroups based on the specific autoantibodies involved. About 90% of patients with generalized MG and 72% with ocular MG have IgG antibodies that bind to the muscle nicotinic acetylcholine receptor (AChR). In comparison, 4–8% have IgG antibodies targeting muscle-specific kinase (MuSK) protein (1, 2). Lipoprotein-related protein 4 (LRP4) antibodies directly block AChR function, and agrin antibodies (Agrin-Ab) are found in some patients with coexisting AChR, MuSK, or LRP4 antibodies (1). These autoantibodies influence clinical symptoms, affect prognosis, and guide treatment options. In generalized MG (gMG), their pathogenic effects reduce functional acetylcholine receptor levels and damage the neuromuscular junction, leading to impaired neuromuscular transmission (3).

Current treatments, including corticosteroids and non-steroidal immunosuppressive therapies (NSISTs), generally suppress the immune system without specifically targeting IgG autoantibodies (4). However, long-term use of corticosteroids is linked to serious side effects such as hyperglycemia, hypertension, osteoporosis, myopathy, and lymphocytopenia (5). Most NSISTs take months to become fully effective and may increase the risk of secondary malignancies, liver dysfunction, and bone marrow suppression (6). Additionally, some gMG patients remain resistant to standard treatments. According to international consensus, refractory gMG is defined by an inadequate response to at least two immunosuppressive therapies, relapse during corticosteroid tapering, or dependence on intravenous immunoglobulin (IVIG) or plasma exchange (PE) (7). These patients often experience frequent myasthenic flares and crises, which frequently lead to hospitalization, emphasizing the need for alternative treatment options (7).

Recent advances in biologic therapies, such as B-cell-depleting agents, complement inhibitors, and FcRn antagonists, highlight the importance of targeting specific immune pathways (8). B cells are crucial in MG development because they produce harmful autoantibodies against acetylcholine receptors (9). B lymphocyte stimulator (BLyS/BAFF) and a proliferation-inducing ligand (APRIL) are cytokines vital for B cell survival and differentiation (10). Telitacicept (Remegen Co., Ltd., China), a biologic targeting B cells, is a recombinant fusion protein that links the transmembrane activator and calcium modulator ligand interactor (TACI) receptor with the Fc domain of human IgG (11). By blocking both BLyS and APRIL, telitacicept inhibits B cell activation and differentiation, offering a potential therapeutic advantage over traditional immunosuppressants.

Approved in China for systemic lupus erythematosus (SLE) in 2021, telitacicept has demonstrated efficacy in immunoglobulin A nephropathy (IgAN), neuromyelitis optica spectrum disorders (NMOSD), Sjögren’s syndrome, and rheumatoid arthritis (11–14). A phase 2 clinical trial in gMG patients reported safety, tolerability, and significant clinical improvement (15). A phase 3 clinical trial (NCT05737160) aimed at further confirming telitacicept’s efficacy for gMG in China was completed in December 2024, but the results have not yet been published. A global phase 3 trial (NCT06456580) is currently ongoing across 54 international sites, including the United States, Canada, and Australia. Telitacicept has received Orphan Drug Designation from both the U.S. Food and Drug Administration (FDA) and the European Medicines Agency (EMA) for the treatment of MG.

Critical considerations include the safety and tolerability of new biologics like telitacicept. Clinical trials show that telitacicept may cause fewer side effects than traditional immunosuppressants, which carry significant long-term risks (16). This is particularly important for MG patients, as many are worried about the side effects of long-term immunosuppressive treatment. Given the unmet need in refractory gMG, this retrospective study looks at the real-world effectiveness of telitacicept in Chinese patients with refractory AChR+ gMG.

## 2 Methods

### 2.1 Study design and intervention

This was a single-center, retrospective study evaluating the real-world effectiveness of telitacicept in patients with refractory AChR+ generalized myasthenia gravis (gMG). All patients received subcutaneous telitacicept at a fixed dose of 160 mg weekly for the first 4 weeks, then 160 mg every two weeks starting from week 5, in line with the dosing regimen approved by China’s National Medical Products Administration (NMPA). Concomitant immunosuppressive therapies were maintained at stable doses unless clinical adjustments were necessary.

### 2.2 Participants

The study included patients treated at the Department of Neurology at the Affiliated Hospital of Qingdao University from July 2023 to July 2024. Eligible patients were adults (aged ≥18 years) with a confirmed diagnosis of generalized myasthenia gravis (gMG). Diagnosis was based on clinical features of fatigable muscle weakness, positive anti-AChR antibody tests via radioimmunoassay, electrophysiological abnormalities (such as repetitive nerve stimulation), and a documented response to acetylcholinesterase inhibitors. Disease severity was classified according to the Myasthenia Gravis Foundation of America (MGFA) classes II–IV (17). Refractory disease status was strictly defined as meeting at least one of the following criteria: inadequate response to two or more immunosuppressive therapies, relapse during corticosteroid dose reduction, or dependence on intravenous immunoglobulin (IVIG) or plasma exchange (PE).

The key exclusion criteria included: receiving IVIG or PE within 2 months before starting telitacicept; using B-cell-depleting agents or other biologics within 6 months before enrollment; having active infections at screening; or having hypersensitivity to biologic products.

All patients provided written informed consent before screening. The study protocol received approval from the Institutional Review Board of the Ethics Committee of the Affiliated Hospital of Qingdao University (Approval No. QYFYEC2024-315).

### 2.3 Outcomes

Demographic and clinical data were systematically gathered, including age at disease onset, disease duration, MGFA classification, anti-AChR antibody levels, and Quantitative Myasthenia Gravis (QMG) scores. Patients underwent standardized monthly assessments over 5 months. The primary outcome was a ≥ 3-point reduction in QMG scores and attainment of MGFA-PIS minimal manifestation status; secondary outcomes included immunosuppressant dose reduction and safety events.

#### 2.3.1 Safety assessment

Adverse events (AEs) were systematically documented using the Common Terminology Criteria for Adverse Events (CTCAE) v5.0 and monitored through scheduled visits and patient reports. All events reported were graded according to CTCAE v5.0.

### 2.4 Statistical analysis

Statistical analyses were conducted using R software (version 4.5.0). Changes from baseline for continuous efficacy outcomes were analyzed with mixed-effects models for repeated measures, and results are presented as least-squares (LS) means with 95% confidence intervals (CI). Cumulative response rates for binary outcomes (such as ≥3-point QMG reduction and MGFA-PIS minimal manifestation status or better) were evaluated using the Kaplan-Meier method, with median time to response and response rates at specific time points reported. Categorical variables were summarized as frequencies and compared using χ² or Fisher’s exact tests. For immunosuppressant usage patterns—including tacrolimus (Tac), mycophenolate mofetil (MMF), and cyclosporine A (CYA)—descriptive statistics are provided due to the small number of users, which limits formal statistical testing. Missing data were handled using last observation carried forward for continuous variables, and patients lost to follow-up were censored in time-to-event analyses. Statistical significance was set at α=0.05, with p-values indicated as *p<0.05, **p<0.01, and ***p<0.001.

## 3 Results

### 3.1 Patient characteristics and disposition

The study included 42 patients with refractory AChR antibody-positive generalized myasthenia gravis (gMG) who started telitacicept treatment between July 2023 and July 2024. The average treatment duration was 3.5 months (range 1–6 months), with most patients (38.1%) undergoing 3 months of treatment. Patient retention rates decreased from 100% at baseline to 90.5% at month 3 and 71.4% at month 5, reflecting real-world treatment patterns.

As shown in **Table 1**, the group was predominantly female (73.8%, n=31), with a median age at disease onset of 56.00 years (IQR 34.75-62.25). Seventeen patients (40.5%) experienced early-onset disease, diagnosed before age 50, while the median disease duration before starting telitacicept was 29.50 months (IQR 13.75-69.75). Disease severity at baseline, classified by MGFA criteria, included 19 patients (45.3%) with class II, 13 (31.0%) with class III, and 10 (23.8%) with class IV involvement. Anti-AChR antibody levels varied widely, with 14.3% (n=6) showing high titers (>20 nmol/L), 14.3% (n=6) with moderate levels (10–20 nmol/L), and 66.7% (n=28) with low levels (<10 nmol/L); antibody data were unavailable for 2 patients (4.8%). Thymic abnormalities (thymoma or hyperplasia) were present in 17 patients (40.5%), Among the entire cohort, 12 patients (28.6%) had a history of thymectomy prior to enrollment, and no patient underwent thymectomy during the telitacicept treatment period. while common comorbidities included hypertension (26.2%, n=11) and thyroid disease (19.0%, n=8). Baseline concomitant therapies reflected the refractory nature of the cohort. Half of the patients (50.0%, n=21) were on corticosteroids with a median dose of 10 mg/day, while pyridostigmine bromide was nearly universal (85.7%, n=36) at a standard dose of 180 mg/day. Non-steroidal immunosuppressants included tacrolimus (23.8%, n=10), mycophenolate mofetil (19.0%, n=8), and cyclosporine A (2.4%, n=1). Notably, 19.0% (n=8) had prior exposure to FcRn antagonists, and 2.4% (n=1) had been treated with complement inhibitors, both discontinued at least 6 months before starting telitacicept.

**Table 1:** Baseline characteristics of the study population (N = 42).

Characteristic	Value
Demographics	
Age at onset, years, median (IRQ)	56.00 (34.75-62.25)
Male	11 (26.2%)
Female	31 (73.8)
Disease features	
Disease duration, months, median (IRQ)	29.50 (13.75-69.75)*
History of thymectomy, n (%)	12 (28.6)
MGFA classification, n (%)	
− Class II	19 (45.3)
− Class III	13 (31.0)
− Class IV	10 (23.8)
AChR-ab titer, nmol/L, n (%)	
− >20	6 (14.3)
− 10-20	6 (14.3)
− <10	28 (66.7)
− Missing	2 (4.8)
Comorbidities, n (%)	
Thymoma/thymic hyperplasia	17 (40.5)
Hypertension	11 (26.2)
Diabetes mellitus	5 (11.9)
Thyroid disease	8 (19.0)
Concomitant medications, n (%)	
Prednisone	21 (50.0)
− Daily dose, mg†	10 (4.5-11.5)*
Pyridostigmine bromide	36 (85.7)
− Daily dose, mg	180 (180-180)*
Tacrolimus	10 (23.8)
Mycophenolate mofetil	8 (19.0)
Cyclosporine A	1 (2.4)
Prior biologic therapies	
FcRn antagonists	8 (19.0)
Complement inhibitors	1 (2.4)

### 3.2 Efficacy outcomes

**Figure 1:**
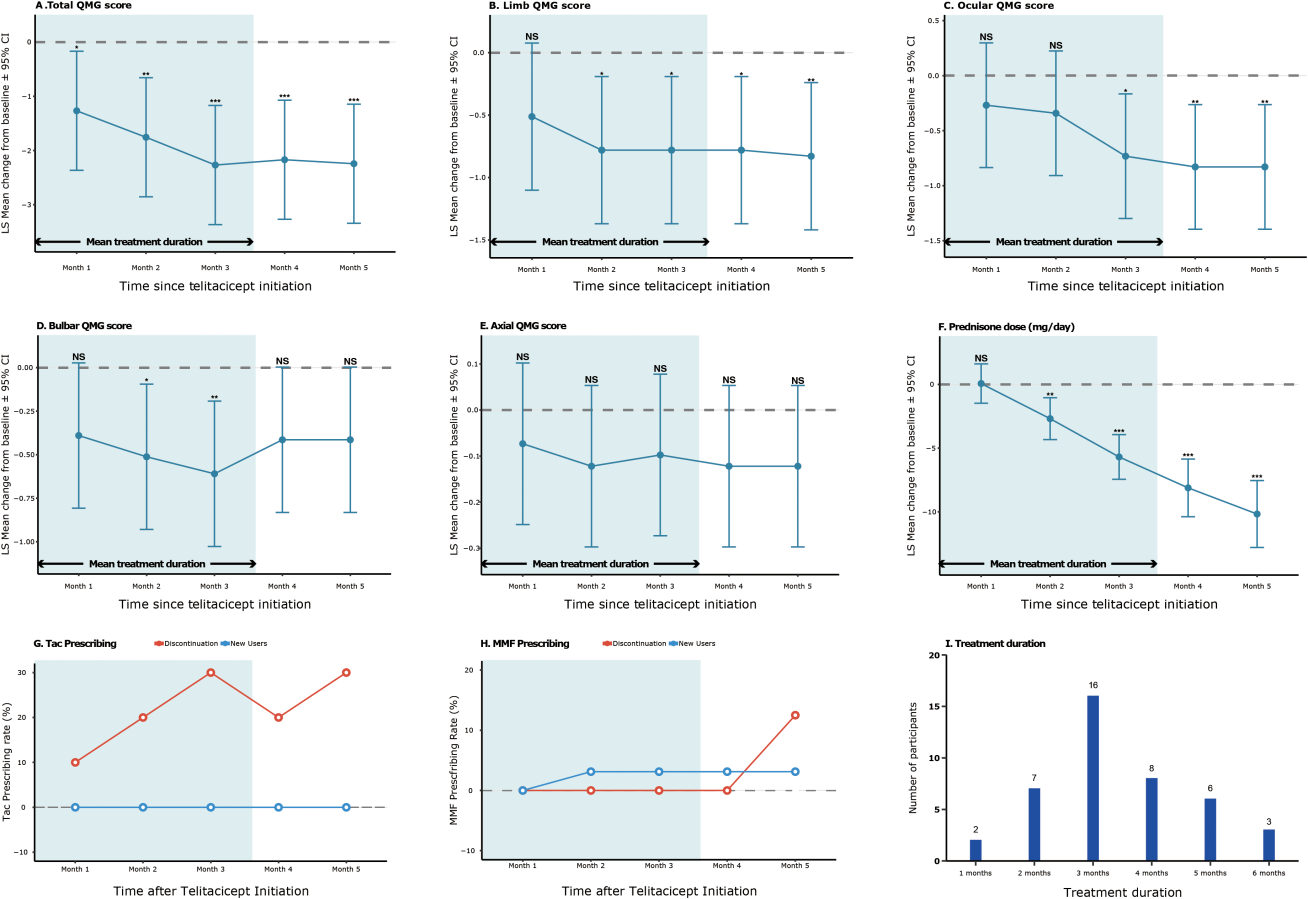
Change from baseline in primary and secondary endpoints. The plots show the changes from baseline in observed scores for total QMG **(A)**, limb QMG **(B)**, ocular QMG **(C)**, bulbar QMG **(D)**, axial QMG **(E)**, and prednisone dose **(F)**. Data points represent the observed least-squares (LS) mean changes from baseline, with error bars indicating 95% CI. QMG = Quantitative Myasthenia Gravis. **(G, H)** illustrate the prescriptions of Tac and MMF before and after telitacicept treatment. **(I)** shows the distribution of participants based on treatment duration. p-values were indicated as *p<0.05, **p<0.01, and ***p<0.001.

Mixed-effects model analysis demonstrated a significant improvement in the QMG total score from baseline to month 5 (LS mean change −2.24, 95% CI −3.34 to −1.15; p < 0.001), with continuous improvement observed throughout the treatment period (**Table 2**). Significant reductions were also observed in limb QMG scores (month 5: −0.83, 95% CI −1.42 to −0.24; p = 0.008) and ocular QMG scores (month 5: −0.83, 95% CI −1.40 to −0.26; p = 0.006) (**Table 2**, **Figures 1A–F**).

**Table 2:** Change from baseline in efficacy outcomes.

Measures		Month 1	Month 2	Month 3	Month 4	Month 5
Total QMG score	Δ (95% CI)	-1.27 (-2.37~-0.17)	-1.76 (-2.85~-0.66)	-2.27 (-3.37~-1.17)	-2.17 (-3.27~-1.07)	-2.24 (-3.34~-1.15)
	p Values	0.028	0.003	<0.001	<0.001	<0.001
Limb QMG score	Δ (95% CI)	-0.51 (-1.10~0.08)	-0.78 (-1.37~-0.19)	-0.78 (-1.37~-0.19)	-0.78 (-1.37~-0.19)	-0.83 (-1.42~-0.24)
	p Values	NS	0.012	0.012	0.012	0.008
Ocular QMG score	Δ (95% CI)	-0.27 (-0.83~0.30)	-0.34 (-0.91~0.22)	-0.73 (-1.30~-0.17)	-0.83 (-1.40~-0.26)	-0.83 (-1.40~-0.26)
	p Values	NS	NS	0.014	0.006	0.006
Bulbar QMG score	Δ (95% CI)	-0.39 (-0.81~0.03)	-0.51 (-0.93~-0.09)	-0.61 (-1.03~-0.19)	-0.41 (-0.83~0.00)	-0.41 (-0.83~0.00)
	p Values	NS	0.020	0.006	NS	NS
Axial QMG score	Δ (95% CI)	-0.07 (-0.25~0.10)	-0.12 (-0.30~0.05)	-0.10 (-0.27~0.08)	-0.12 (-0.30~0.05)	-0.12 (-0.30~0.05)
	p Values	NS	NS	NS	NS	NS
Pred dose (mg/day)	Δ (95% CI)	0.07 (-1.48~1.61)	-2.69 (-4.33~-1.04)	-5.69 (-7.45~-3.94)	-8.12 (-10.38~-5.85)	-10.17 (-12.80~-7.54)
	p Values	NS	0.002	<0.001	<0.001	<0.001
PB dose (mg/day)	Δ (95% CI)	0.17 (-8.88~9.22)	-8.19 (-18.03~1.66)	-27.10 (-37.93~-16.26)	-13.17 (-27.35~1.01)	-17.35 (-34.05~-0.65)
	p Values	NS	NS	<0.001	NS	0.044

**Figure 2:**
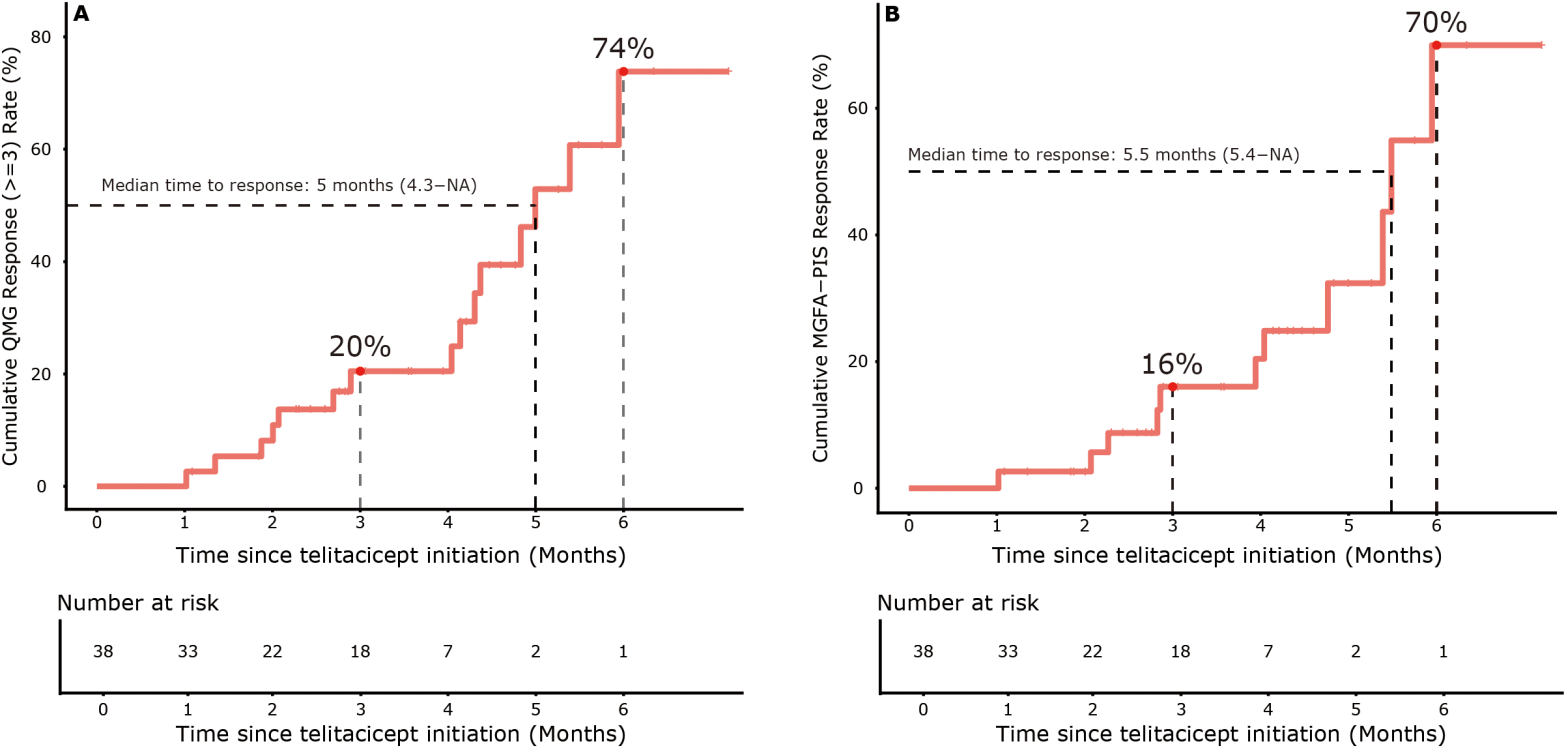
The plots display the cumulative QMG response (Δ≥3) rate **(A)** and cumulative MGFA-PIS response rate **(B)**, with median time to response (95% CI) and cumulative response rates by 3 and 6 months.

Kaplan-Meier analysis showed cumulative response rates of 69.9% (95% CI 16.2–89.2) for MGFA-PIS minimal manifestation status and 73.8% (95% CI 29.1–90.3) for QMG improvement (a ≥3-point reduction) at 6 months (**Figure 2**). The analysis included 38 patients at risk at baseline, with numbers decreasing over time due to study discontinuation (23 at month 3, 3 at month 6), consistent with the protocol, which did not impute missing outcome data for patients lost to follow-up. The median time to response was 5.5 months for MGFA-PIS and 5 months for QMG improvement. Response rates gradually increased from 16% at month 3 to 69.9% at month 6 for MGFA-PIS and from 20.5% to 73.8% for QMG improvement during the same period (**Figures 2A, B**).

Among patients who previously did not respond to other biologic therapies (n=9), including complement inhibitors or FcRn antagonists, 55.6% (5/9) achieved minimal manifestation status with telitacicept, indicating its effectiveness in multiple refractory cases.

### 3.3 Reductions in concomitant medications

Significant reductions in concomitant medications were observed. The median daily prednisone dose in the 3–6 months prior to telitacicept initiation was 10.0 mg (IQR 5.0-20.0; data available for n=31). Prednisone doses decreased by the least squares mean of −10.17 mg/day (95% CI −12.80 to −7.54; p < 0.001) at month 5, while pyridostigmine bromide doses decreased by −17.35 mg/day (95% CI −34.05 to −0.65; p = 0.044). Among patients using non-steroidal immunosuppressants at baseline, discontinuation rates reached 30% for tacrolimus and 12.5% for mycophenolate mofetil by month 5, with few new starts observed (**Figures 1G, H**). The only patient who used CYA at baseline discontinued treatment during the study, with no new CYA administrations observed.

### 3.4 Safety

Safety analyses reported TEAEs in 6 patients (14.3%), with details summarized in **Table 3**. There were no Grade 3–4 events or treatment discontinuations due to adverse effects. The most common TEAEs were upper respiratory infections (7.1%) and injection site reactions (4.8%). Telitacicept proved tolerable, with no treatment-related allergic reactions, serious infections, or adverse events requiring dose adjustments.

**Table 3:** TEAEs during telitacicept therapy (N = 42).

Preferred term	All grade, n (%)	≥Grade 3, n (%)
Infections		
Upper respiratory infection	3 (7.1)	0 (0)
Urinary tract infection	1 (2.4)	0 (0)
General disorders		
Fatigue	2 (4.8)	0 (0)
Injection site reaction	2 (4.8)	0 (0)
Nervous system disorders		
Headache	1 (2.4)	0 (0)

## 4 Discussion

Managing refractory generalized myasthenia gravis (gMG) remains a significant therapeutic challenge, as demonstrated by the limited responses to standard immunosuppressive treatments in our group. Our real-world study shows that telitacicept provides notable clinical benefits in refractory AChR+ gMG, with strong improvements confirmed through mixed-effects model analysis. The least-squares mean reduction in the QMG total score of -2.24 points (95% CI: -3.34 to -1.15) at month 5 indicates meaningful clinical improvement, consistent with previous evidence from phase 2 trials (15). Our findings are particularly relevant given the strict refractory criteria used, which require failure of at least 2 immunosuppressive therapies or dependence on rescue treatments, thereby identifying a population with exceptionally high unmet needs (7).

The treatment regimen of 160 mg weekly for four weeks, followed by biweekly doses, proved pharmacologically sound, aligning with the drug’s pharmacokinetic profile, which shows an elimination half-life of 10.9-12.5 days and sustained target engagement (18). This dosing strategy led to gradual clinical improvement, with response rates increasing (from 16% to 69.9% for MGFA-PIS and from 20.5% to 73.8% for QMG improvement between months 3 and 6), indicating that a longer treatment duration could provide greater benefits. The significant reductions across multiple QMG domains—especially in ocular (LS mean -0.83, p=0.006) and limb scores (LS mean -0.83, p=0.008) at month 5—demonstrate comprehensive neuromuscular improvement. Although the effect size was slightly smaller than that in phase 2 trials (-7.7-point reduction) (15), this probably reflects key differences between controlled trials and real-world practice, including broader patient selection and varying concomitant therapies in our cohort.

Kaplan-Meier analysis revealed significant response rates: 69.9% of patients reached MGFA-PIS minimal manifestation status, and 73.8% achieved QMG improvement (≥3-point reduction) within 6 months, demonstrating the progressive effects of telitacicept. These meaningful improvements enabled substantial corticosteroid reduction, with LS mean prednisone doses decreasing by -10.17 mg/day (95% CI -12.80 to -7.54, p<0.001) at month 5, approaching the <5 mg target recommended by international guidelines (19, 20). The discontinuation rates of non-steroidal immunosuppressants (30% for tacrolimus, 12.5% for mycophenolate mofetil) further highlight telitacicept’s potential as a steroid-sparing treatment. Our findings align with recent real-world evidence indicating telitacicept’s effectiveness in refractory gMG (21, 22).

The consistent treatment responses across various demographic and clinical subgroups, including age, disease duration, MGFA class, and antibody levels, demonstrate broad applicability in refractory gMG. Interestingly, five of nine patients who previously did not respond to complement inhibitors or FcRn antagonists achieved minimal manifestation status with telitacicept, highlighting the importance of targeting alternative pathways in treatment-resistant cases. This observation supports the emerging paradigm of mechanism-based therapeutic selection in the management of MG (8, 23, 24).

The favorable safety profile of telitacicept aligns with phase 2 trial observations (15), with mostly mild injection-related reactions and infections being the most common TEAEs. The mixed-effects model analysis used in our study, which accounts for within-patient correlations and missing data, provides more reliable estimates of treatment effects than traditional methods, improving the robustness of our findings.

Several limitations should be considered when interpreting these findings. The retrospective, single-center design presents potential selection and information biases, while the modest sample size (n=42) restricts subgroup analyses. The limited number of patients using specific non-steroidal immunosuppressants hampers our ability to perform formal statistical testing for these agents, leading to a mainly descriptive presentation. The declining number of patients at risk (from 38 at month 1 to 3 at month 6) illustrates real-world challenges but warrants caution in interpreting long-term estimates. Financial constraints that lead to treatment discontinuation in some patients may affect the generalizability of the results. Additionally, the average treatment duration of 3.5 months, though reflective of real-world patterns, might underestimate the potential benefits of longer-term therapy.

These results are influenced by the ongoing global development of telitacicept, including its FDA and EMA orphan drug designations for MG and the multinational phase 3 trial (NCT06456580). While our study provides promising real-world evidence, prospective controlled trials with longer follow-up are needed to define telitacicept’s role in the MG treatment algorithm clearly. Future research should also explore biomarkers that predict response and identify the most effective combination strategies with current therapies.

## 5 Conclusion

This study provides clinically relevant evidence that telitacicept improves measurable outcomes in refractory AChR+ gMG across different patient subgroups, while also enabling a reduction in concomitant immunosuppression. The consistency of these real-world findings with earlier clinical trial data strengthens the therapeutic rationale for BLyS/APRIL inhibition in MG treatment. While awaiting confirmatory phase 3 results, our experience indicates that telitacicept is a valuable addition to the limited options available for this challenging patient population.

## Data Availability

The raw data supporting the conclusions of this article will be made available by the authors, without undue reservation.

## References

[1] Gilhus NE Verschuuren JJ . Myasthenia gravis: subgroup classification and therapeutic strategies. Lancet Neurol. (2015) 14:1023–36. doi: 10.1016/S1474-4422(15)00145-3, PMID: 26376969

[2] Shelly S Paul P Bi H Dubey D Milone M Sorenson EJ . Improving accuracy of myasthenia gravis autoantibody testing by reflex algorithm. Neurology. (2020) 95:e3002–11. doi: 10.1212/WNL.0000000000010910, PMID: 32938782

[3] Engel AG Arahata K . The membrane attack complex of complement at the endplate in myasthenia gravis. Ann New York Acad Sci. (1987), 326–32. doi: 10.1111/j.1749-6632.1987.tb51301.x, PMID: 3318619

[4] Skeie GO Apostolski S Evoli A Gilhus NE Illa I Harms L . Guidelines for treatment of autoimmune neuromuscular transmission disorders. Eur J Neurol. (2010) 17:893–902. doi: 10.1111/j.1468-1331.2010.03019.x, PMID: 20402760

[5] Huscher D Thiele K Gromnica-Ihle E Hein G Demary W Dreher R . Dose-related patterns of glucocorticoid-induced side effects. Ann Rheum Dis. (2009) 68:1119–24. doi: 10.1136/ard.2008.092163, PMID: 18684744

[6] Machkhas H Harati Y . Side effects of immunosuppressant therapies used in neurology. Neurol Clin. (1998) 16:171–88. doi: 10.1016/S0733-8619(05)70373-X, PMID: 9421547

[7] Suh J Goldstein JM Nowak RJ . Clinical characteristics of refractory myasthenia gravis patients. Yale J Biol Med. (2013) 86:255–60., PMID: 23766745 PMC3670444

[8] Iorio R . Myasthenia gravis: the changing treatment landscape in the era of molecular therapies. Nat Rev Neurol. (2024) 20:84–98. doi: 10.1038/s41582-023-00916-w, PMID: 38191918

[9] Menon D Barnett C Bril V . Novel treatments in myasthenia gravis. Front Neurol. (2020) 11:538. doi: 10.3389/fneur.2020.00538, PMID: 32714266 PMC7344308

[10] Kowalczyk-Quintas C Schuepbach-Mallepell S Vigolo M Willen L Tardivel A Smulski CR . Antibodies that block or activate mouse B cell activating factor of the tumor necrosis factor (TNF) family (BAFF), respectively, induce B cell depletion or B cell hyperplasia. J Biol Chem. (2016) 291:19826–34. doi: 10.1074/jbc.M116.725929, PMID: 27451394 PMC5025672

[11] Fan Y Gao D Zhang Z . Telitacicept, a novel humanized, recombinant TACI-Fc fusion protein, for the treatment of systemic lupus erythematosus. Drugs Today (Barc). (2022) 58:23–32. doi: 10.1358/dot.2022.58.1.3352743, PMID: 35107091

[12] Lv J Liu L Hao C Li G Fu P Xing G . Randomized phase 2 trial of telitacicept in patients with IgA nephropathy with persistent proteinuria. Kidney Int Rep. (2023) 8:499–506. doi: 10.1016/j.ekir.2022.12.014, PMID: 36938094 PMC10014376

[13] Ding J Jiang X Cai Y Pan S Deng Y Gao M . Telitacicept following plasma exchange in the treatment of subjects with recurrent neuromyelitis optica spectrum disorders: A single-center, single-arm, open-label study. CNS Neurosci Ther. (2022) 28:1613–23. doi: 10.1111/cns.13904, PMID: 35851754 PMC9437241

[14] Shi F Xue R Zhou X Shen P Wang S Yang Y . Telitacicept as a BLyS/APRIL dual inhibitor for autoimmune disease. Immunopharmacol Immunotoxicol. (2021) 43:666–73. doi: 10.1080/08923973.2021.1973493, PMID: 34519594

[15] Yin J Zhao M Xu X Zhang M Xu Z Li Z . A multicenter, randomized, open-label, phase 2 clinical study of telitacicept in adult patients with generalized myasthenia gravis. Eur J Neurol. (2024) 31:e16322. doi: 10.1111/ene.16322, PMID: 38726639 PMC11235933

[16] Ebrahim Soltani Z Rahmani F Rezaei N . Autoimmunity and cytokines in Guillain-Barré syndrome revisited: review of pathomechanisms with an eye on therapeutic options. Eur Cytokine Netw. (2019) 30:1–14. doi: 10.1684/ecn.2019.0424, PMID: 31074417

[17] Barohn RJ . Standards of measurements in myasthenia gravis. Ann N Y Acad Sci. (2003) 998:432–9. doi: 10.1196/annals.1254.056, PMID: 14592911

[18] Xie J Fan X Su Y Zhou H Cao S Zhu X . Pharmacokinetic characteristics, safety, and tolerability of telitacicept, an injectable recombinant human B-lymphocyte stimulating factor receptor-antibody fusion protein, in healthy Chinese subjects. Clin Pharmacol Drug Dev. (2022) 11:1273–83. doi: 10.1002/cpdd.1136, PMID: 35844038 PMC9796261

[19] Murai H . The Japanese clinical guidelines 2022 for myasthenia gravis and Lambert-Eaton myasthenic syndrome: an overview. Brain Nerve. (2024) 76:7–12. doi: 10.11477/mf.1416202551, PMID: 38191133

[20] Wiendl H Abicht A Chan A Della Marina A Hagenacker T Hekmat K . Guideline for the management of myasthenic syndromes. Ther Adv Neurol Disord. (2023) 16:17562864231213240. doi: 10.1177/17562864231213240, PMID: 38152089 PMC10752078

[21] Zhang Z Wang Z Du X Huang X Zhang Y . Refractory generalized myasthenia gravis treated successfully with telitacicept: two cases report. J Neurol. (2024) 271:584–8. doi: 10.1007/s00415-023-12036-y, PMID: 37804335

[22] Lin J Li Y Gui M Bu B Li Z . Effectiveness and safety of telitacicept for refractory generalized myasthenia gravis: a retrospective study. Ther Adv Neurol Disord. (2024) 17:17562864241251476. doi: 10.1177/17562864241251476, PMID: 38751755 PMC11095194

[23] Jaretzki A 3rd Barohn RJ Ernstoff RM Kaminski HJ Keesey JC Penn AS . Myasthenia gravis: recommendations for clinical research standards. Task Force of the Medical Scientific Advisory Board of the Myasthenia Gravis Foundation of America. Ann Thorac Surg. (2000) 70:327–34. doi: 10.1016/S0003-4975(00)01595-2, PMID: 10921745

[24] Hehir MK Hobson-Webb LD Benatar M Barnett C Silvestri NJ Howard JF Jr . Rituximab as treatment for anti-MuSK myasthenia gravis: Multicenter blinded prospective review. Neurology. (2017) 89:1069–77. doi: 10.1212/WNL.0000000000004341, PMID: 28801338

